# Systems Approach to Identify Common Genes and Pathways Associated with Response to Selective Serotonin Reuptake Inhibitors and Major Depression Risk

**DOI:** 10.3390/ijms20081993

**Published:** 2019-04-23

**Authors:** Ankit Srivastava, Priyanka Singh, Hitesh Gupta, Harpreet Kaur, Neha Kanojia, Debleena Guin, Mamta Sood, Rakesh Kumar Chadda, Jyoti Yadav, Divya Vohora, Luciano Saso, Ritushree Kukreti

**Affiliations:** 1 Genomics and Molecular Medicine Unit, Institute of Genomics and Integrative Biology (IGIB), Council of Scientific and Industrial Research (CSIR), Delhi 110007, India; ankit.srivastava@igib.in (A.S.); priyanka3811053@gmail.com (P.S.); guptah96@gmail.com (H.G.); neha.kanojia@igib.in (N.K.); debleena.guin@igib.in (D.G.); j.yadav@igib.in (J.Y.); 2 Department of Psychiatry, All India Institute of Medical Sciences, Ansari Nagar, New Delhi 110029, India; soodmamta@gmail.com (M.S.); drrakeshchadda@gmail.com (R.K.C.); 3 Department of Pharmacology, School of Pharmaceutical Education and Research, Jamia Hamdard, New Delhi 110062, India; 4 Academy of Scientific and Innovative Research (AcSIR), CSIR-Institute of Genomics and Integrative Biology (CSIR-IGIB) Campus, New Delhi 110007, India; 5 Department of Bioinformatics, Delhi Technological University, Shahbad Daulatpur, Main Bawana Road, Delhi 110042, India; 6 Genomic Medicine Institute, Lerner Research Institute, Cleveland Clinic Foundation, 9500 Euclid Avenue, Cleveland, OH 44195, USA; kaurh2@ccf.org; 7 Department of Physiology and Pharmacology “Vittorio Erspamer”, Sapienza University of Rome, P. le Aldo Moro 5, 00185 Rome, Italy

**Keywords:** MDD, SSRI, antidepressant, cushing syndrome, axon guidance, glutamatergic synapse, cAMP signaling pathway, GWAS

## Abstract

Despite numerous studies on major depressive disorder (MDD) susceptibility, the precise underlying molecular mechanism has not been elucidated which restricts the development of etiology-based disease-modifying drug. Major depressive disorder treatment is still symptomatic and is the leading cause of (~30%) failure of the current antidepressant therapy. Here we comprehended the probable genes and pathways commonly associated with antidepressant response and MDD. A systematic review was conducted, and candidate genes/pathways associated with antidepressant response and MDD were identified using an integrative genetics approach. Initially, single nucleotide polymorphisms (SNPs)/genes found to be significantly associated with antidepressant response were systematically reviewed and retrieved from the candidate studies and genome-wide association studies (GWAS). Also, significant variations concerning MDD susceptibility were extracted from GWAS only. We found 245 (Set A) and 800 (Set B) significantly associated genes with antidepressant response and MDD, respectively. Further, gene set enrichment analysis revealed the top five co-occurring molecular pathways (*p* ≤ 0.05) among the two sets of genes: Cushing syndrome, Axon guidance, cAMP signaling pathway, Insulin secretion, and Glutamatergic synapse, wherein all show a very close relation to synaptic plasticity. Integrative analyses of candidate gene and genome-wide association studies would enable us to investigate the putative targets for the development of disease etiology-based antidepressant that might be more promising than current ones.

## 1. Introduction

Major depressive disorder (MDD) is the third largest cause of burden of disease and it is responsible for almost 80% of psychiatric hospitalizations. According to recently conducted world mental health surveys, MDD is experienced by 10–15% people in their lifetime [1] and it can lead to high incidence of suicide. Over 800,000 lives are lost yearly due to suicide, which translates to 3000 suicide deaths every day [2]. It is almost more than half a century now since the first antidepressant drug was discovered, starting from non-specific monoamine oxidase inhibitors (MAO-I) and tricyclic antidepressants (TCA) to target specific selective serotonin reuptake inhibitors (SSRIs). Among them, SSRIs have proven to be the most effective drugs to date, yet approximately 30–40% of depressive patients do not or partially respond to the therapy whereas 60–75% fail to achieve complete remission [3]. This may be attributed to the poor understanding of MDD pathophysiology and lack of etiology-based drugs. Candidate gene-based studies and GWAS have shown that the clinical heterogeneity in therapeutic outcome is also influenced by a variety of genetic (single nucleotide polymorphisms, SNP; copy number variations, CNV; insertions, I; deletions, D etc.), pathophysiological and environmental factors [4,5].

With the advent of high throughput technologies it became possible to generate numerous genomic datasets to identify genetic markers associated with complex disorders and predict drug response. However, these datasets are not consistent enough to turn these findings into clinical practice. The inconsistency may be attributed to the difference in ethnicity of the studied population, underpowered study design, and various confounding factors like age of onset, the severity of disease, gender, etc. Therefore, there is an impending need for integrating these datasets so that we can have a more advance understanding of disease etiology as well as drug response. In pursuit of a better understanding of the problem, several integrated approaches have been put forward for studying the interactions between disease-associated genes and proteins [6]. Network and pathway analysis of candidate genes involved in MDD has provided important information about gene interaction and regulation in MDD [7]. A very recent study has explored the common link for pathogenesis between MDD and glioblastoma using the transcriptomics convergence method, thus providing a new approach to analyze the available huge genetic datasets [8].

With the genetic data on SSRI response and MDD available across diverse populations, a systematic review will help us to better interpret (or curate) the findings of these studies. Therefore, in this study, we first systematically reviewed the literature concerning the genetics of SSRI response (studies on responder versus non-responder patients on SSRIs) and MDD (studies on MDD cases versus healthy controls). Further, we used an integrative genetics approach and retrieved common genes and pathways involved in SSRI response and MDD manifestation to find out potential drug targets for etiology-specific antidepressants development. We identified 29 overlapping genes from a systematic literature search concerning SSRI response and MDD development. Finally, we performed functional enrichment analysis using the WEB-based GEne SeT AnaLysis Toolkit (WebGestalt) [9] (http://webgestalt.org/) to identify the top pathways that are inter-linked between antidepressants response outcome as well as MDD, on the molecular basis. Hence, by integrating genes associated with disease and drug response studies we found a list of genes and pathways which can be validated and might be novel molecular targets for etiology-based antidepressant development.

## 2. Results

A systematic review of candidate gene studies for SSRI response and systematic literature search of GWAS for SSRI response and MDD both revealed 245 and 800 genes to be significantly associated with SSRI response and MDD pathophysiology, respectively. Using these sets of genes, we retrieved commonly associated genes, pathways, and gene ontology (GO) terms, between SSRI response and MDD.

### 2.1. Systematic Search and Study Selection of Antidepressant Response Candidate Gene Studies

**Figure 1:**
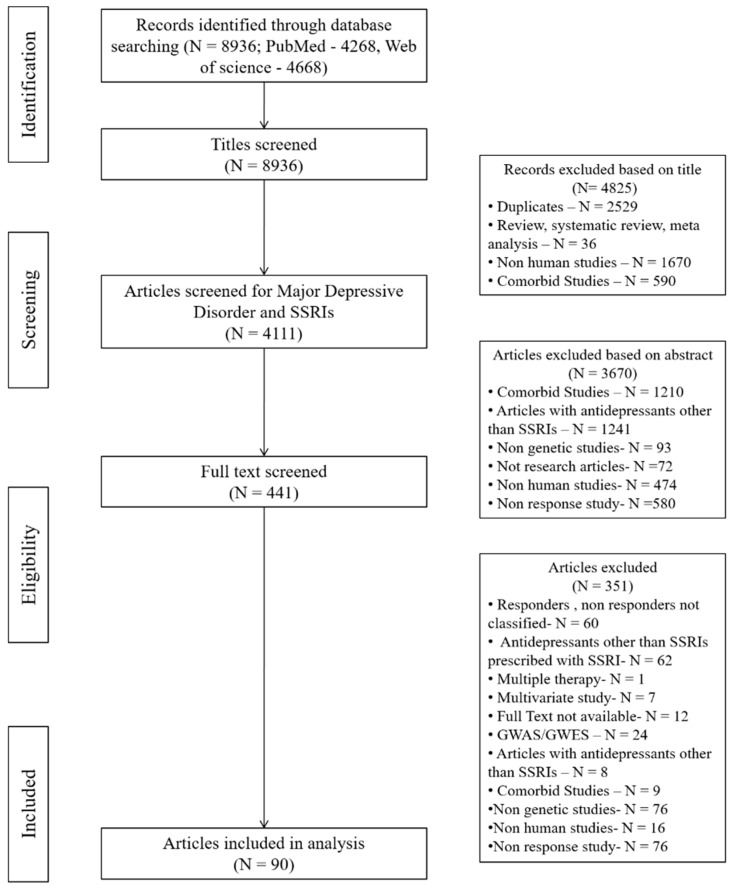
PRISMA flowchart of the study selection process. Reasons for articles that were excluded given in the diagram.

Systematic search strategy for identifying SSRI response associated genes extracted 8936 candidate gene studies (4268 from MEDLINE and 4668 from Web of Science), which were further reduced to 441 unique articles of relevance after the title and abstract screening. Among these excluded articles, 2529 were duplicate articles between MEDLINE and Web of Science. Further, 2144 non-human, 1800 co-morbid, 580 non-response, 1241 non-SSRIs studies, and 201 other articles including non-genetic studies, review articles, genome-wide studies, editorials, and letters were also removed. Remaining 441 articles were then searched for their full text and 351 articles were again excluded from the study, as they did not meet the inclusion criteria (Figure 1), leading to a final of 90 articles for data extraction and processing.

### 2.2. Data Extraction from Candidate Gene Studies of SSRI Response

All the 186 significantly associated SNPs (*p-*value ≤ 0.05) from the 90 selected articles are summarized in Table 1 (complete table in Table S1 online). Sample size along with their phenotype and drugs studied in each of the articles are also detailed in the same. In some research studies, authors had performed two types of analysis, one, responder versus non-responder and second, remitter versus non-remitter; here, we have tabulated all the significant SNPs reported in either kind of analysis.

### 2.3. Data Extraction from GWAS of SSRI Response and MDD Susceptibility

Genes related to SSRI response and MDD etiology were also retrieved from the genome-wide literature. We retrieved 144 articles in total, comprising GWAS with keywords “Depression” or “Depressive Disorder”. Among them, seven GWAS [67,68,69,70,71,72,73] were related to SSRI response (Table 2) and eight GWAS [74,75,76,77,78,79,80,81] were related to MDD etiology (Table 3). In total, 423 and 3884 SNPs with *p-*value ≤ 0.0001 were found to be associated with SSRI response and MDD, respectively (Tables S3 and S4 online).

### 2.4. Data Processing and Genetic Co-Occurrence of SSRI Response and MDD

**Figure 2:**
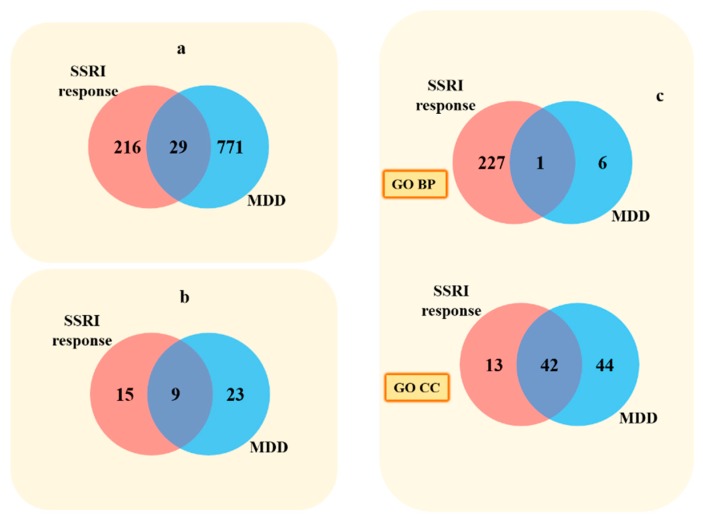
Common Genes (**a**), KEGG biological pathways (**b**), and GO terms (**c**) among SSRI response and MDD.

A total of 186 significant SNPs from a systematic review of SSRI candidate gene studies and 423 SNPs from GWAS of SSRI response were merged to form the first data, Set A, representing SSRI response. Additionally, 3884 SNPs from GWAS of MDD formed the second data, Set B, relating to MDD pathophysiology. Beside this, both sets of SNPs were enriched with probable genetic variations found to be in LD (r^2^ = 1) using the HaploReg v4.1 online tool. Now enriched Set A and Set B comprise 718 and 4769 SNPs, respectively. Enriched Set A and B thus obtained were mapped to genes using the SCAN and Ensembl VEP online tools. After excluding unmapped genes, pseudogenes, orfs, miRNAs, and uncharacterized genes, these annotated genes were assigned their respective HGNC IDs. This obtained a pool of 245 and 800 genes associated with SSRI response and MDD etiology, respectively (Table S5 online). Furthermore, we identified the overlap between SSRI-response genes (Set A) with that of MDD genes (Set B) and found 29 common genes. Since all the MDD associated genes were extracted from GWAS articles, i.e., an unbiased source, therefore, all of the 29 overlapped genes can be considered as unbiased molecular players co-occurring between SSRI response and MDD susceptibility (Figure 2a).

### 2.5. Functional Enrichment of SSRI Response and MDD Gene Pool

**Figure 3:**
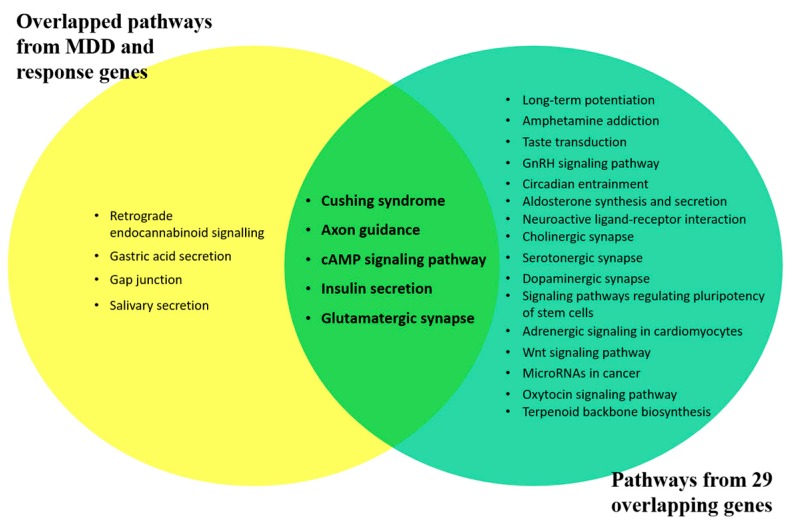
Top five pathways associated with an antidepressant response as well as MDD etiology.

The WebGestalt web-tool was used to further enrich the genes obtained from systematic literature mining. The GO term and pathway enrichment analysis were performed keeping default GO slim classification, 0.05 significance level, and the minimum number of genes in a category was set at 3. The significance value was adjusted by the false discovery rate (FDR) analysis using the Benjamini–Hochberg (BH) procedure (Tables S7 and S8 online). Set A of the SSRI-response genes were significantly enriched in 298 GO terms, involving 228 GO biological process terms, 15 GO molecular function terms, and 55 GO cellular component terms along with 24 KEGG pathway terms. Set B of the MDD etiology genes was significantly enriched in 94 GO terms, involving 7 GO biological process terms, 1 GO molecular function terms, and 86 GO cellular component terms along with 32 KEGG pathway terms. On overlapping, these results showed nine common KEGG pathways (Table S6 online) and 43 common GO terms as shown in Figure 2b,c. This overlap directs a common molecular ground between the mechanism behind antidepressant response and MDD pathophysiology. Since the biased data source (candidate study available literature) in Set A was overlapped with Set B which has an unbiased source (GWAS), common enriched biological pathways must be unbiased and can be used as putative future novel drug targets for MDD. Moreover, functional enrichment of overlapping gene set, i.e., Set C pinpoints the significant pathways involved in both antidepressant response and MDD etiology. Altogether, 5 out of 21 enriched pathways, namely, Cushing syndrome (*p* = 0.005), Axon guidance (*p* = 0.007), cAMP signaling pathway (*p* = 0.010), Insulin secretion (*p* = 0.016), and Glutamatergic synapse (*p* = 0.028) were coinciding when compared to nine commonly enriched pathways among Set A and B (Figure 3). Thus, these represents the most promising pathways, which on in- vitro or in vivo validation, may prove to be new drug targets for etiology-based antidepressant therapy. Thereafter, we evaluated genes commonly present in the five enriched pathways, for their possible roles or interactions in both antidepressant response and MDD pathophysiology, from both the data Sets A and B. Thus, the top recognized genes were: *KCNK2, CACNA1C, CAMK2D, GSK3B, APC, CRHR2, CRHR1, PDE11A, ADCY9, CREB5, ADCY3, GNAI3, DVL3, ADCY2, TCF7L1, RAP1B, WNT1, CACNA1I* (Cushing syndrome); *SLIT3, CAMK2D, GSK3B, RND1, SEMA5A, SEMA6D, GNAI3, ABLIM1, SRGAP3, EFNA5, SEMA3E, NTNG1, BMPR2, EPHB6, ROBO1, BOC, ROBO2* (Axon guidance); *GRIA4, CACNA1C, CAMK2D, HTR1A, ADRB1, DRD2, GRIA3, BDNF, HTR1B, TSHR, ATP1B2, ADCY9, ABCC4, CREB5, ADCY3, GNAI3, ADCY2, RAP1B, ACOX3, PLD2* (cAMP signaling pathway); *CACNA1C, CAMK2D, CHRM3, ATP1B2, ADCY9, CREB5, ADCY3, ADCY2, PCLO* (Insulin secretion); *GRIA4, GRM7, CACNA1C, GRIK2, GRIA3, SLC17A7, ADCY9, ADCY3, KCNJ3, GNAI3, ADCY2, PLD2* (Glutamatergic synapse).

## 3. Discussion

Major depressive disorder is a progressive brain disease with one of the leading cause of disability-adjusted life years affecting approximately 10–15% of the population worldwide [82]. Various treatment regimens are present for MDD, but SSRI pharmacotherapy is being commonly used and often recommended as a first-line treatment option in moderate-to-severe depression because of their higher efficacy and lower side effects compared to other antidepressants. Majorly, all the antidepressants available up to date are aimed at symptomatic management rather than complete cure owing to poorly understood MDD disease etiology. Since the response to antidepressant treatment varies markedly between individuals due to considerable clinical heterogeneity, therefore, the role of genetic predictors of antidepressant response and MDD disease *per se* are of utmost importance for the development of better treatment regimen which would further improve clinical management of MDD.

In this study, we used an integrative genetics approach to unbiasedly elucidate the possible association between antidepressant therapy and MDD etiology. Initially, an extensive literature search was done for identification of genetic variants involved in antidepressant response and disease etiology, followed by functional gene set enrichment, and thereby distinguishing the GO terms and molecular pathways involved in both antidepressant response and MDD susceptibility. For the first time in this uniquely designed study, to the best of our knowledge, has incorporated the amalgam of two exhaustive datasets of disease, concerning its pathophysiology and drug response genes, in search of precise drug targets to accelerate future drug development with minimal side effects.

For quality assessment of the studies included in this systematic review, based on the modified criteria used by Guin D et al. [83], the present study has incorporated the most comprehensive quality assessment scoring of research articles for screening SSRI response articles. Our systematic review has retrieved a total of 55 genes from the 90 research articles which were found to be involved in various synaptic transmission and neuronal development pathways. For instance, rs1083801 (*GRM7*, a glutamate receptor gene), was found to be most significantly associated with the early response with SSRIs [54]. The *TPH2* gene and its variant, rs4760815, has been reported as probable risk factors for the development of MDD and also associated with SSRI response [84]. In addition, other genetic variants from *TPH2*, (rs11179027 and rs17110532); glutamate receptor ionotropic, kainate 2 gene, *GRIK2* (rs543196), glutamic acid decarboxylase gene, *GAD1* (rs3828275) and *SLC6A4* (rs2066713) were also reported to be strongly associated with SSRI response [15]. SERTPR often regarded as 5-HTTLPR is a serotonin transporter gene promoter polymorphism which is been exhaustively studied in both remission rate and response rate of antidepressants [85]. A review and meta-analysis study by Kato and Serretti [86] has extensively studied and reported a significant association between the 5-HTTLPR variant and better response to antidepressants. Zill et al. [87] have identified functional polymorphism in the β1 adrenergic receptor 1165G>C (rs1801253), which was found to be involved in conferring the faster response towards antidepressant treatment, but did not influences the depressive phenotype. Furthermore, *HTR2A*, a serotonergic receptor gene variant rs7997012 is found to be associated with SSRI response as well as remission [56,59,88,89]. However, Illi A et al. [90] and Kishi T et al. [28] have reported a negative association of rs7997012 with SSRI response or remission. Thus, to overcome such inconsistencies across several research studies, we need an innovative approach to eliminate the biases and limitation of candidate gene studies [91]. Hence in the present study, we have opted for “candidate gene studies and GWAS overlapping” approach to overcome the lack-of-harmony among candidate gene studies. As a result, the systematic review of candidate gene studies and GWAS was merged to ascertain the unbiased genes and molecular pathways involved in both antidepressant response and MDD etiology.

After merging findings from the systematic review and GWAS studies, all the genetic variants were grouped into Set A and Set B followed by their LD enrichment, gene annotation, and functional enrichment analysis, to reveal multiple relevant pathways among antidepressant response and MDD etiology (Figure 1). Furthermore, 56 KEGG pathways (24 from Set A and 32 from Set B) were identified with 9 commonly enriched pathways, namely Cushing syndrome, Retrograde endocannabinoid signaling, Axon guidance, cAMP signaling pathway, Glutamatergic synapse, Insulin secretion, Gap junction, Gastric acid secretion, and Salivary secretion, from Set A and Set B. In the view of MDD etiology, the most interesting molecular pathways are Axon guidance, Glutamatergic synapse, and Cushing syndrome. These 9 co-occurring pathways were further overlapped with the molecular pathways enriched from the 29 common genes implicated in antidepressant response and MDD etiology. This results in five overlapping molecular pathways, namely, Cushing syndrome, Axon guidance, cAMP signaling pathway, Insulin secretion, and Glutamatergic synapse. As MDD is a complex disorder, multiple pathways are expected to be involved in its etiology and our result is in concordance with the same showing the interplay of stress, neurodevelopmental, synaptic plasticity, and metabolic physiology in MDD etiology and antidepressant response.

Cushing syndrome, as being the top pathway of the analysis, is a metabolic disorder caused by overproduction of cortisol (glucocorticoid) produced by the adrenal cortex of the adrenal gland in response to glucocorticoids medication or in stressful condition. The adrenal gland is a part of stress-responsive hypothalamic pituitary adrenal (HPA) axis which consists of stimulating and feedback mechanism involving the hypothalamus, pituitary, and adrenal gland, and thus regulates the production of glucocorticoids. The HPA axis dysregulation has been implicated in the pathophysiology of many neuropsychiatry traits including MDD [92,93,94]. Genes associated with the homeostatic response to environmental stressors particularly lies in the HPA axis [95].

Corticotrophin-releasing hormone (CRH), released by the hypothalamus, stimulates the release of adrenocorticotropic hormone (ACTH) from pituitary which in turns regulate the levels of cortisol secreted from the adrenal gland. Hence CRH and its downstream effects are of prime importance which is controlled by cortisol level through feedback mechanism and two CRH receptors i.e., *CRHR1* and *CRHR2* [96]. Alteration in *CRHR1* and *CRHR2* activity may lead to HPA dysregulation and can be a major risk factor for depressive symptoms. Bradley et al. [97] demonstrated the role of genetic variants in *CRHR1* as moderators of the effects of child abuse on adult depressive symptoms in two independent populations which was further confirmed by a replication study which reported the association of TAT (rs7209436, rs110402, and rs242924) haplotype in *CRHR1* in predicting the adult depression [98,99]. Woody et al. [100] also supported the hypothesis of protective *CRHR1* haplotype (TAT) as their result suggested the development of brooding among children without the protective *CRHR1* haplotype. Moreover, animal studies also indicate the prenatal glucocorticoid exposure can cause epigenetic instability in *CRHR1* promoter which further increases the risk for the affective disorder in offspring’s across two generations [101]. Epistatic interaction between *AVPR1b*, *CRHR1,* and *BDNF* genes has also been reported to be involved in susceptibility to MDD [102,103]. Ressler et al. [104] reported the involvement of interaction of 5-HTTLPR S allele with *CRHR1* haplotype in predicting adult depression in individuals with child abuse. The SNPs rs110402, rs242924 rs3779250, rs7209436, and rs173365 from *CRHR1* and *CRHR2* genes were reported to be positively associated with MDD in the Japanese population [105]. However, a *CRHR2*-based 10 SNP study showed no significant allelic or genotypic differences among unipolar patients and matched healthy controls [106].

In addition, there are evidences for an antidepressant response via glucocorticoid receptors (GRs) where it has been demonstrated that sertraline increases human hippocampal neurogenesis via a GR-dependent mechanism [107]. A recent study has shown the significant association of rs41423247 polymorphism with fluoxetine response in depressed patients [10]. Another study of haplotype-tag SNPs (rs1876828, rs242939 and rs242941) in *CRHR1* shown the significantly improved response to antidepressants among highly anxious patients homozygous for the GAG haplotype, suggesting the possible role of *CRHR1* and other stress-inflammatory pathway genes in variable antidepressant response [108,109].

Increasing evidence suggests the dysfunction of glutamatergic neurotransmission impairs neuroplasticity in the brain and this leads to major depressive disorder [110,111]. Many novel targets in relation to this pathway are evidenced for causing the depression phenotype. In the past decade, *NMDA* has gained the utmost attention with respect to the biology of depression and also serves as a potential target for drug development and treatment. As under pathological condition, elevated levels of glutamate resulted in the impairment of synaptic plasticity and even excitotoxicity. Alternate depression hypothesis includes exposure to stress inculpates the release of the high level of stress hormone i.e., cortisol from the adrenal gland. Correspondingly, adequate literature demonstrates that stress and glucocorticoids are responsible for alteration in the expression and activity of vesicular proteins of the neurotransmission of glutamate [112]. Emerging evidences from post-mortem studies has reported the dysregulation of genes and increased glutamate levels, thus highlighting the role of altered glutamate signaling in MDD patients [113]. Decrease expression of presynaptic genes in MDD patients such as *SYN3*, *SNAP25*, essential for vesicular release of neurotransmitters have been reported. Likewise, a significant downregulation of postsynaptic genes was also reported in the dentate gyrus (DG) and CA1 of MDD patients such as *AMPA* receptors, specifically *GLU1* and *GLU3* [114]. In the same note, several *NMDA* gene variations have been reported to have an association with an abnormality in *NMDA* receptors. One such study had shown significant association of *GRIN1* (rs4880213) with depression. Further studies revealed that variations in the *GRIN2B* were associated with schizophrenia, psychiatric disorders, and brain plasticity [115]. Similarly, downregulation of *NMDA* receptor subunits *GRIN1A* and *GRIN2B*, as well as *PSD-95* have been demonstrated in the anterior prefrontal cortex of MDD subjects [116]. Likewise, metabotropic glutamate receptor 7 (*GRM7*) encodes the protein mGluR7 mediates the glutamate neurotransmission, found to be involved in the development of the major depressive disorder. Li W et al. [117] studied the association of genetic variation of the *GRM7* gene with MDD and schizophrenia and reported the significant association of *GRM7* gene variation (rs779706) with MDD and (rs2229902 and rs9870680) with schizophrenia in the Han Chinese population. Thus, it is hypothesized that dysfunction of ionotropic and metabotropic receptors are associated with the depressed phenotype. However, in a resequencing study, none of the *GRM7* variants had shown the significance level with MDD in the Dutch cohort [118].

Also, a number of studies support the association between the antidepressant response and *GRIK4* in MDD patients [89,119,120]. Genes encoded ionotropic glutamate receptors were studied with respect to citalopram treatment and demonstrated a significant association of two SNPs (rs4825476 and rs2518224) located within *GRIA3* and *GRIK2,* respectively, with the treatment-emergent suicidal ideation in MDD patients [121]. Moreover, in order to explore the potential targets for treatment-resistant depressive patients, emerging evidence from clinical trials supported the use of glutamate receptor modulators for the treatment of depression and these include non-competitive *NMDA* receptor antagonists such as ketamine, subunit (*NR2B*)-specific *NMDA* receptor antagonists, *NMDA* receptor glycine-site partial agonists and metabotropic glutamate receptor (mGluR) modulators [122]. Therefore, glutamate pathways and its associated receptors are important and further insights and detailed understanding could help us to target the accurate site for future drug development.

Neuronal circuit formation involves a molecular cascade of events such as axon guidance, where axons move to their target cells in a complex, constantly changing the environment. It has been speculated that change in this gene-environment interaction may lead to alteration in axon guidance followed by its implication in neuropsychiatric disorders. Furthermore, a meta-analysis by S Jovanova O et al. [123], has reported three methylated sites associated with depressive symptoms and were also found to be involved in axon guidance as a pathway in major depression. Interestingly, research articles have also shown that miRNAs implicated in axon guidance are involved in differential antidepressant response where miRNAs namely miR-146a-5p, miR-146b-5p, miR-221-3p, miR-24-3p, miR-26a-5p are known to be involved in axon guidance and were also associated with antidepressant response in MDD patients [124,125]. Our current finding of Slit guidance ligand 3 gene, *SLIT3* in antidepressant and MDD gene set is in consensus with findings from Glessner JT et al. [126], where they have performed genome-wide copy number variation scan of large cohort of MDD patients and controls and has observed 5q35.1 as the most significant locus harboring the *SLIT3* gene which is integral to repulsive axon guidance. Thus synaptic plasticity mediated via axon guidance is a topic of new research and can further be studied for better antidepressant development.

Since the majority of intracellular messenger cascades are regulated either by G protein-coupled receptors (GPCRs) or protein tyrosine kinases (PKAs) and are major activator of various cellular and molecular signaling pathways such as cortisol secretion in the adrenal cortex contributing to Cushing’s syndrome and neurotransmitters such as serotonin and norepinephrine, which are known to mediate the effects of antidepressant treatments modulates secondary messenger cascades via interaction with GPCRs or PKAs [127,128]. Hence, receptor activation induced by ligands such as neurotransmitters confers to cAMP generation via stimulating adenylate cyclase (AC), and their binding to the G-protein subtype leads to activation of PKA, which is an important factor for driving several biological functions either by phosphorylation or dephosphorylation of specific target proteins, undermines the antidepressant actions. Furthermore, the cAMP response element binding protein (*CREB*), a transcription factor that mediates the actions of the cAMP cascade, is a substrate for PKA, is involved in regulating gene expression, and has the capability to modulate their transcriptional activity, which is important for cellular adaptions during antidepressant administration.

Dowlatshahi et al. [129] have reported low *CREB* levels in post-mortem temporal cortex of naive major depressive disorder patients as compared to MDD patients treated with antidepressants. Odagaki et al. [130] have even demonstrated the increased immunoreactivities of phosphorylated *CREB* as well as total *CREB* levels in the prefrontal cortex of depressed suicide victims and specifically in antidepressant drug-free subjects. Also, with a slight trend for increased levels of PKA-Cα in depressed suicide victims only as compared to healthy controls. As a downstream consequence, the expression of various target genes critical to the organization of neuronal networks and synaptic plasticity, like neurotrophin, brain-derived neurotrophic factor (*BDNF*), and neuropeptide Y (*NPY*) is also increased contributing towards antidepressant-mediated changes in structural remodeling, neuronal plasticity and synaptic restructuring [131,132]. Henceforth, evidence from such studies confer the ability of HTR receptors in either stimulating or inhibiting the AC-cAMP-PKA signaling transduction pathway but further invokes solicitation and functional validation of pathway genes in undermining the action of antidepressants, as this transduction pathway is highly regulated by several other factors such as stress, apoptosis, inflammation, and others.

Hence pathway enrichment analysis of “biased and unbiased” merged data is assured to facilitate our understanding of the underlying molecular mechanism of the complex trait of anti-depressant response and major depression as a disease, to circumvent the symptomatic respite and design a definite therapy. Application of this elaborated analytical tactics in translational research concerning complex disorders like MDD would be beneficial after in-vitro and in-vivo validation of the top promising pathways and genes involved in antidepressant response and MDD etiology.

## 4. Methodology

**Figure 4:**
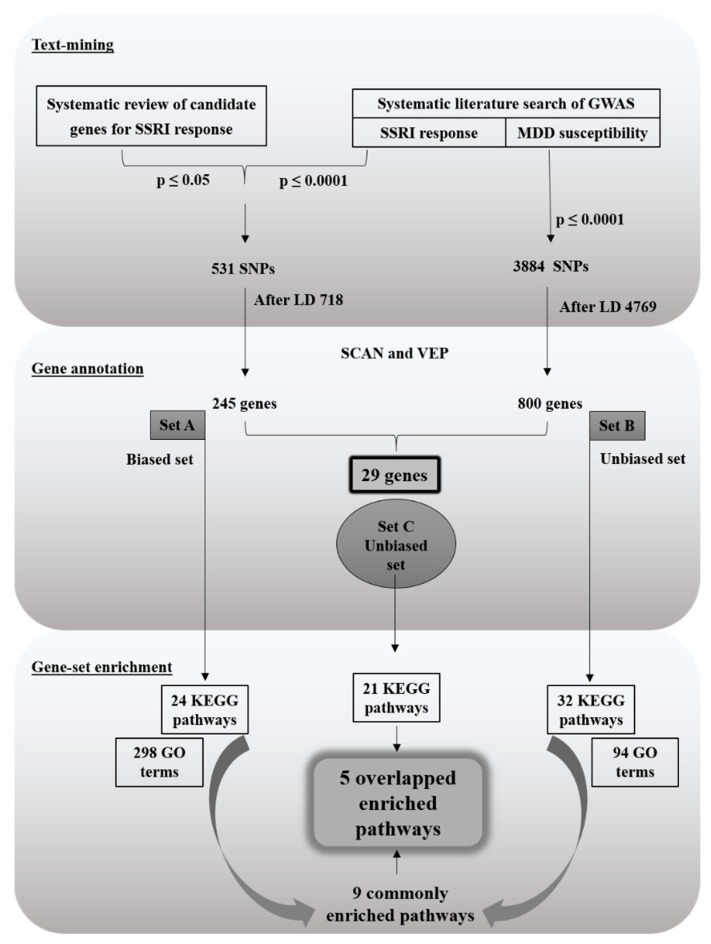
Flow chart of the study.

The complete workflow of the study is represented in Figure 4. We initiated with integrating significantly associated SNPs from candidate genes studies and GWAS concerning SSRI response and from GWAS of MDD etiology. Therefore all the SNPs from candidate gene studies and GWAS of SSRI response were merged in one group and another group consist significant genetic variations from MDD GWAS. Further, they were annotated into genes followed by functional and pathway enrichment analysis.

### 4.1. A systematic Review of Antidepressant Response Candidate Gene Studies

Among currently available antidepressants, selective serotonin reuptake inhibitors (SSRIs) like escitalopram, sertraline, and fluoxetine are the most commonly prescribed drugs [133] considering their higher efficacy and lower side-effects. Hence in the present study, we have considered “SSRIs” and “antidepressants” interchangeably in this manuscript. A systematic literature search was performed in accordance with PRISMA guidelines [134]. The MEDLINE and Web of Science databases were searched using Medical Subject heading (MeSH) terms “selective serotonin reuptake inhibitor”, “SSRI”, “pharmacogenetics”, “pharmacogenomics”, “response”, “treatment outcome”, “SNP”, “variant”, “polymorphism” with AND/OR Boolean operators to extract all the studies evaluating association of SNPs with SSRI treatment outcome in patients with MDD. The search and study selection was carried out independently by three authors (AS, HG, and PS) covering the articles published till 28th February 2018.

The searches were confined to human and English language studies. All the articles that were review, meta-analysis, commentary, editorial, clinical trial, letter, randomized control trial, technical report were excluded. Articles were sorted for their relevance at two stages, first using the title and second using their abstract. At the first stage of title screening, duplicates, reviews, systematic reviews, meta-analysis, non-human, and co-morbid studies were removed. Secondly, the abstracts of all remaining articles were retrieved and screened based on the inclusion and exclusion criteria of the study. Inclusion criteria required genetic association of SSRI response in MDD patients, with age range of 18–75 years, and that response should be inferred based on gold standard severity rating scales like Hamilton Depression Rating Scale (HAM-D) or Montgomery Asberg Depression Rating Scale (MADRS). Whereas studies involving MDD as co-morbidity or MDD patients with other severe medical illness, psychiatric disorder or substance abuse were excluded. Detailed exclusion criteria are given in Figure 1.

### 4.2. Data Extraction and Quality Assessment of Antidepressant Response Candidate Gene Articles

Data, from selected full-text articles, were extracted by AS, HG, and PS and checked by DG and RK. Ethnicity, used response criteria, sample size, genes with information of genetic variant, related genotypic or allelic frequency and respective reported *p*-value, drug, dose, and follow-up period were included in the data collection table. Ethnicity was classified as reported in the respective article, else the country in which the study was conducted, assumed to be the individual’s ethnicity. A cut-off *p*-value ≤ 0.05 was used for extracting SNPs from the selected articles. Methodological quality of each article was assessed by two independent reviewers (AS and HG) using modified criteria for quality assessment, as used by Guin D et al. [83]. The quality assessment was scored on 8 parameters (Table S2 online), with a positive score awarded for each detail present in the study, the lack of detail was described as 0. Conflicting scores were reached to a consensus upon discussing with DG and RK. If the score obtained was 7 or higher, the study was considered as high quality.

### 4.3. A Systematic Literature Search of GWAS of SSRI Response and MDD Susceptibility

Data were also extracted from GWAS, using keywords “Depression” or “Depressive Disorder”, correlating genetic variability with SSRI response in patients with MDD, and distinguishing MDD patients from healthy controls. To maintain homogeneity in studies, only those articles were selected where researchers have opted peripheral blood as their source of DNA extraction. In the case of disease vs. control GWAS, the diagnosis has been made by a psychiatrist/clinical psychologist, otherwise, a study has been excluded from the pool. Similarly, in GWAS correlating SNPs with SSRI response, if the response were adjudicated using standard depression severity rating scales the study was included, else excluded. Studies considering meta-analysis were also excluded. A cut-off *p-*value ≤ 0.0001, was used for extracting SNPs reported in respective GWAS.

### 4.4. Data Processing of Candidate Gene Studies for SSRI Response and GWAS of SSRI Response and MDD Susceptibility

SNPs from candidate and GWAS studies were retrieved and categorized into two data sets, Set A containing SNPs associated with SSRI response (data from both systematic review and GWAS of SSRI response), and Set B comprising SNPs associated with MDD susceptibility (data only from GWAS of MDD disease). Here, we were investigating the targets for etiology based antidepressant development using genomic integration of disease and drug response, one dataset needed to be unbiased and hence we decided not to include candidate gene studies for MDD. Thus, Set A can be considered to be a biased data set as it contains SNPs from candidate gene studies as well as from GWAS, whereas Set B is genetically unbiased data set as it contains data from GWAS only. As interpatient genetic variability can modulate SSRI clinical response and MDD etiology, therefore, in order to widen the genetic region to find out the biological relevance of the associated SNPs, we have incorporated all the probable genetic variations found to be in linkage disequilibrium (LD) with previously extracted SNPs of Set A and Set B. Furthermore, pairwise LD among the extracted SNPs of set A and B were performed using HaploReg v4.1 which uses mammalian conservation algorithm from GERP and SiPhy-omega with LD threshold (r^2^) = 1, corresponds to 100% LD [135]. SNPs that are found to be in 100% LD in all the four major population i.e., African, American, Asian, and European were included in the respective SNP sets. In addition, SCAN (SNP and CNV Annotation Database) [136] (http://www.scandb.org/newinterface/about.html) and VEP (Variant Effect Predictor —Ensembl) [137] (https://asia.ensembl.org/info/docs/tools/vep/index.html) online tools were employed for gene annotation for all the identified SNPs, which were extracted and enriched from LD. The SCAN utilizes two ways for SNP annotation, i.e., relative position based and eQTLs (expression quantitative trait loci) method. Relative position-based method identifies a gene based on SNP position (intronic, inter-genic, etc.) or an intergenic variant can be annotated to a gene if it is in LD with any other variant present in the gene. An eQTLs-based method annotates a gene whose quantitative expression is altered by input SNP. Whereas VEP annotates an SNP to a gene if it has a functional effect on that gene, transcript, protein sequence or regulatory regions. The SNPs, which remained unmapped, were excluded from further analysis. Genes thus obtained were assigned HGNC (HUGO Gene Nomenclature Committee) (https://www.genenames.org/) IDs manually [138]. The pseudogenes, hypothetical loci, non-coding RNAs, non-protein coding genes, open reading frames (orfs), microRNA (miRNA), and uncharacterized genes were excluded from each dataset. Further, a third data set, Set C, was also framed which contained all the overlapping genes from Set A and Set B. Set C was used to distinguish the most significant pathways from the commonly enriched pathways among Set A and Set B.

### 4.5. Functional Enrichment Analysis

For each of the three datasets, functional enrichment analysis was conducted using a WEB-based GEne SeT AnaLysis Toolkit (WebGestalt) [9]. Gene Ontology (GO) term enrichment analysis (http://www.geneontology.org/) and pathway enrichment analysis with Kyoto Encyclopedia of Genes and Genomes (KEGG) engine (https://www.genome.jp/kegg/) was performed keeping default GO slim classification, 0.05 significance level and a minimum number of genes in a category was set at 3. For enrichment analysis of each dataset, the significance value was adjusted by the false discovery rate (FDR) analysis using the Benjamini–Hochberg (BH) procedure. The GO terms and molecular pathways overlapping between Set A and Set B were extracted, which further overlaid with the enriched pathways from Set C to identify pathophysiological grounds which can be targeted to develop etiology based antidepressants.

## 5. Conclusions

Burgeoning literature evidence has so far pointed out the clinical relevance of antidepressant response and MDD etiology individually, but such studies are not consistent enough to manifest these findings into clinical practice. In the present study, we have focused on highlighting the possible confounding factors responsible for antidepressant response and MDD pathophysiology altogether. Thus we have shortlisted significant genes and pathways implicated in the same which can be further utilized as novel molecular targets for the development of more efficacious antidepressant drugs. Application of this elaborated analytical approach in translational research concerning complex disorders like MDD would be beneficial after in-vitro and in-vivo validation.

## Figures and Tables

**Table 1:** Characteristics of included studies for assessment of the association between genetic variants and SSRIs response in major depressive disorder.

No.	Study Name	Sample Size					Genes	Studied Variants	*p*-Value		OR (95% CI) Genotypic	OR (95% CI) Allelic	Drugs	FP	Score
		M	F	Total (*n*)	Responder/Remitter (*n*)	Non Responder/Non Remitter (*n*)			Genotypic	Allelic					
1	Nouraei H et al. (2018) [10]	33	67	100	70	30	*GCR*	rs41423247	0.008	0.032	3.3 (1.35–8.09)	2.2 (1.09–4.44)	Fluoxetine	6	9
2	Firouzabadi N et al. (2017) [11]	25	75	100	33	67	*ADRB1*	rs1801253	0.003	0.0002	5.7 (1.4–23.9)	3.3 (1.72–6.50)	Sertraline	6	9
3	Xu Z et al. (2016) [12]	116	165	281	114	50	*TPH2*	rs11178998	0.0209	N.A.	2.3 (1.14–4.50)	N.A.	SSRI	6	9
								rs7963717	0.0239	N.A.	2.2 (1.09–4.35)				
4	Manoharan A et al. (2016) [13]	35	67	102	56	46	*SLC6A4*	5-HTTLPR	0.0066	N.A.	4.0 (1.45–11.03)	N.A.	Fluoxetine	6	9
5	Paroni G et al. (2017) [14]	95	234	329	176	153	*KL*	rs9536314	0.011	N.A.	N.A.	N.A.	Escitalopram, Sertraline, Paroxetine, Citalopram	22	8
6	Lim SW et al. (2014) [15]	59	180	239	154	85	*TPH2*	rs4760815	0.00001	N.A.	N.A.	N.A.	SSRI	6	9
								rs11179027	0.00002	N.A.					
								rs17110532	0.00009	N.A.					
								rs17110747	0.0002	N.A.					
							*GRIK2*	rs543196	0.00005	N.A.					
								rs572487	0.0001	N.A.					
							*GAD1*	rs3828275	0.00007	N.A.					
								rs12185692	0.0002	N.A.					
							*SLC6A4*	rs2066713	0.0001	N.A.					
								rs2020942	0.0003	N.A.					
7	Fukui N et al. (2014) [16]	65	58	123	24	35	*COMT*	rs2075507	N.A.	0.0036	N.A.	N.A.	Fluvoxamine	12	7
								rs1544325	N.A.	0.0036					
								rs5993883	N.A.	0.015					
8	Li X et al. (2014) [17]	141	149	290	220	70	SLC17A7	rs74174284	0.014	0.008	0.57 (0.38–0.87)	N.A.	SSRI	6	9
9	Wang XC et al. (2014) [18]	109	189	298	219	79	*BDNF*	rs6265	0.001	N.A.	N.A.	N.A.	Paroxetine	6	9
							*GDNF*	rs2973049	0.005						
								rs2216711	0.005						
10	Han KM et al. (2013) [19]	13	81	94	37	19	*CYP2D6*	rs1065852	0.001	0.001	N.A.	N.A.	Escitalopram	12	9
11	Shima Sahraian et al. (2013) [20]	26	78	104	65	39	*SLC6A4*	5-HTTLPR	0.023	N.A.	N.A.	N.A.	Citalopram	14	8
12	Liu Z et al. (2013) [21]	72	113	185	98	87	*PDLIM5*	rs2433320	0.0145	N.A.	N.A.	N.A.	Fluoxetine	6	9
13	Mitjans M et al. (2013) [22]	35	120	155	96	51	*CNR1*	rs806368	0.029	0.021	N.A.	N.A.	Citalopram	12	9
								rs806371	0.045	0.016					
								rs806377	0.188	0.043					
14	Myung W et al. (2013) [23]	34	54	88	46	42	*SLC6A4*	5-HTTLPR	0.004	N.A.	N.A.	N.A.	Sertraline, Fluoxetine	6	9
15	Wang Y et al. (2012) [24]	182	221	403	287	78	*DRD2*	rs2734833	0.0445	N.A.	N.A.	N.A.	SSRI	6	8
16	Yang Z et al. (2012) [25]	182	221	403	130	35	*APC*	rs2229992	N.A.	0.05	N.A.	N.A.	SSRI	6	8
							*SRP19*	rs495794		0.0011					
							*REEP5*	rs153549		0.0015					
								rs153560		0.0009					
17	Xu Z et al. (2012) [26]	121	187	308	114	52	*HTR1B*	rs6298	0.023	N.A.	N.A.	0.39 (0.17–0.91)	SSRI	6	9
18	Illi A et al. (2011) [27]	36	49	85	29	56	*SLC6A4*	5-HTTLPR	0.03	N.A.	N.A.	N.A.	Citalopram, Fluoxetine, Paroxetine	6	9
19	Kishi T et al. (2010) [28]	121	144	265	150	115	*HTR2A*	rs1928040	0.054	0.0252	N.A.	N.A.	Fluvoxamine, Sertraline, Paroxetine	8	7
20	Kishi T et al. (2010) * [28]	121	144	265	150	115	*HTR2A*	rs1928040	0.0910	0.0418	N.A.	N.A.	Fluvoxamine, Sertraline, Paroxetine	8	7
21	Liou YJ et al. (2009) [29]	186	263	449	42	117	*KCNK2*	rs6667764	0.046	0.360	N.A.	N.A.	Citalopram, Fluoxetine	8	9
								rs10494994	0.05	0.082					
								rs6686529	0.019	0.0008					
22	Min W et al. (2009) [30]	272	307	579	243	119	*SLC6A4*	5-HTTLPR	0.032	0.617	N.A.	N.A.	SSRI	6	9
23	Kishi T et al. (2009) [31]	60	61	121	60	61	*CLOCK*	rs3736544	0.0043	0.0026	N.A.	N.A.	Fluvoxamine	8	9
24	Kishi T et al. (2009) * [31]	60	61	121	60	61	*CLOCK*	rs3736544	0.0065	0.0026	N.A.	N.A.	Fluvoxamine	8	9
								rs3749474	0.073	0.025					
25	Tsai SJ et al. (2009) [32]	208	300	508	126	61	*TPH2*	rs2171363	0.009	0.512	N.A.	N.A.	Fluoxetine, citalopram	8	7
								rs4290270	0.019	0.459					
26	Arias B et al. (2009) [33]	114	33	147	96	52	*DTNBP1*	rs760761	0.03	0.007	N.A.	N.A.	Citalopram	4	8
27	Wong ML et al. (2008) [34]	37	71	108	-	-	*CYP3A4*	rs2242480	N.A.	0.02	N.A.	N.A.	Fluoxetine	8	7
							*PSMD13*	rs3817629		0.04					
							*CD3E*	rs2231449		0.002					
							*PRKCSH*	rs160841		0.02					
							*PSMA7*	rs2057169		0.004					
								rs2057168		0.003					
								rs2281740		0.002					
								rs3746651		0.01					
28	Tsai SJ et al. (2008) [35]	101	129	230	74	92	*GSK3B*	rs334558	0.002	0.02	N.A.	N.A.	Fluoxetine, Citalopram	4	8
								rs13321783	0.002	0.002					
								rs2319398	0.011	0.011					
29	Gau YT et al. (2008) [36]	100	128	228	74	43	*NGFR*	rs2072446	0.039	0.012	N.A.	N.A.	Fluoxetine, Citalopram	8	9
30	Bozina N et al. (2008) [37]	69	61	130	65	65	*SLC6A4*	5-HTTLPR	0.005	0.0004	N.A.	N.A.	Paroxetine	6	9
31	Papiol S et al. (2007) [38]	35	124	159	95	51	*CRHR2*	rs2270007	0.018	0.002	N.A.	N.A.	Citalopram	4	8
32	Ham BJ et al. (2007) * [39]	29	76	105	42	63	*TPH1*	rs1800532	0.047	0.017	N.A.	N.A.	Citalopram	8	9
33	Choi MJ et al. (2006) [40]	24	59	83	57	26	*COMT*	rs6265	0.012	0.009	N.A.	N.A.	Citalopram	8	7
34	Hong CJ et al. (2006) [41]	93	131	224	81	143	*HTR1A*	rs6295	0.009	N.A.	N.A.	N.A.	Fluoxetine	4	8
							*SLC6A4*	5-HTTLPR	0.001						
35	Choi MJ et al. (2005) * [42]	51	20	71	22	49	*HTR2A*	rs6311	0.018	0.034	N.A.	N.A.	Citalopram	4	8
36	Kraft JB et al. (2005) [43]	49	47	96	77	19	*SLC6A4*	rs25531	N.A.	0.03	N.A.	N.A.	Fluoxetine	12	9
37	Yu YW et al. (2003) * [44]	67	90	157	4	115	*IL-1B*	rs193922490	0.028	N.A.	N.A.	N.A.	Fluoxetine	4	7
38	Yoshida K et al. (2002) [45]	22	32	54	35	19	*SLC6A4*	5-HTTLPR	0.059	0.01	N.A.	N.A.	Fluvoxamine	6	9
39	Yin L et al. (2016) [46]	141	151	290	220	70	*DRD4*	rs1800544	0.03	0.41	N.A.	N.A.	Fluoxetine, paroxetine, sertraline, citalopram	6	9
40	Hun Soo Chang et al. (2011) [47]	16	99	115	49	25	*BDNF*	rs6265	0.001	0.006	N.A.	N.A.	Escitalopram	8	9
41	Lin KM et al. (2010) * [48]	36	205	241	69	102	*CYP1A2*	rs4646425	0.002	0.03	2.3 (1.12–4.73)	N.A.	Paroxetine	8	9
								rs2472304	0.024	0.01	0.39 (0.19–0.82)				
								rs2470890	0.015	0.004	0.34 (0.16–0.74)				
42	Lee SH et al. (2010) * [49]	17	47	64	35	29	*MRP1*	rs2239330	0.038	0.005	N.A.	N.A.	Citalopram	8	9
								rs212087	0.194	0.052					
								rs212090	0.133	0.035					
43	Tsai SJ et al. (2009) [50]	138	196	334	101	52	*COMT*	rs4680	0.02	0.006	N.A.	N.A.	Fluoxetine, citalopram	8	9
44	Yu YW et al. (2006) [51]	94	128	222	83	139	*HTR1A*	rs6295	0.007	N.A.	N.A.	N.A.	Fluoxetine	4	8
45	Suzuki Y et al. (2004) [52]	29	23	52	35	17	*HTR1A*	rs1800042	0.042	N.A.	N.A.	N.A.	Fluvoxamine	12	9
46	Jamerson BD et al. (2013) [53]	44	60	104	55	49	*MTRR*	rs1801394	0.0077	N.A.	N.A.	N.A.	SSRI	12	8
							*MTHFR*	rs1801131	0.0313						
47	Fabbri C et al. (2013) [54]	598	943	1541	260	1281	*GRM7*	rs1083801	0.0000005	N.A.	N.A.	N.A.	Citalopram	2	8
							*GRIK2*	rs599545	0.0003						
								rs2786247	0.0008						
								rs2852584	0.0002						
								rs2518313	0.0003						
								rs2786239	0.0006						
							*GRIA4*	rs495498	0.0008						
								rs10791773	0.0009						
								rs994575	0.0009						
								rs11226856	0.0002						
							*PRKCE*	rs505310	0.0005						
							*CAMK2D*	rs12508566	0.0009						
48	Glubb DM et al. (2010) [55]	N.A.	N.A.	285	47	19	*ADM*	rs11042725	0.001	N.A.	N.A.	N.A.	Paroxetine	6	6
49	Peters EJ et al. (2009) [56]	746	1207	1953	N.A.	N.A.	*HTR2A*	rs1923884	N.A.	0.02	N.A.	0.75 (0.58–0.97)	Citalopram	6	9
								rs7997012	N.A.	0.0002	N.A.	1.43 (1.13–1.81)			
50	Peters EJ et al. (2009) * [56]	746	1207	1953	N.A.	N.A.	*HTR2A*	rs1923884	N.A.	0.01	N.A.	0.72 (0.55–0.95)	Citalopram	6	9
								rs7997012	N.A.	3.0 × 10^−5^	N.A.	1.52 (1.20–1.95)			
51	Mrazek DA et al. (2009) * [57]	443	631	1074	1042	32	*SLC6A4*	SERTin2	0.041	N.A.	N.A.	N.A.	Citalopram	6	9
								5-HTTLPR	0.039						
52	Kato M et al. (2006) [58]	44	56	100	57	23	*SLC6A4*	5-HTTLPR	0.043	N.A.	N.A.	N.A.	Paroxetine, Fluvoxamine	6	8
53	McMahon FJ et al. (2006) * [59]	748	1205	1953	N.A.	N.A.	*HTR2A*	rs7997012	0.00004	2.0 × 10^−5^	N.A.	N.A.	Citalopram	6	9
								rs1928040	0.0701	0.0446					
54	McMahon FJ et al. (2006) [59]	748	1205	1953	N.A.	N.A.	*HTR2A*	rs7997012	2.0 × 10^−6^	4.0 × 10^−5^	N.A.	N.A.	Citalopram	6	9
								rs1928040	0.0149	0.0709					
55	Peters EJ et al. (2004) [60]	47	49	96	77	19	*SLC614*	rs25533	0.037	N.A.	0.33 (0.08–1.35)	N.A.	Fluoxetine	12	7
56	Ji Y et al. (2012) [61]	N.A.	N.A.	1232	541	691	*COMT*	rs13306278	N.A.	0.04	N.A.	0.78 (0.62–0.99)	SSRI	6	7
								rs9332381	N.A.	0.006		1.71 (1.16–2.51)			
57	Lekman M et al. (2008) [62]	748	1205	1953	954	415	*FKBP5*	rs4713916	0.0027	0.0007	N.A.	N.A.	Citalopram	6	7
58	Lekman M et al. (2008) * [62]	748	1205	1953	723	466	*FKBP5*	rs4713916	0.042	0.042	N.A.	N.A.	Citalopram	6	7
59	Kraft JB et al. (2007) [63]	735	1179	1914	991	669	*SLC6A4*	rs25533	0.05	N.A.	1.81 (0.92–3.56)		Citalopram	6	9
60	Binder EB et al. (2010) [64]	746	1207	1953	982	726	*CRHBP*	rs10473984	0.0068	0.0044	N.A.	1.42 (1.11–1.81)	Citalopram	6	9
								rs10474485	0.018	0.0065		1.25 (1.06–1.46)			
								rs10055255	0.020	0.017		1.19 (1.04–1.39)			
							*CRHR2*	rs2267716	0.024	0.013		1.20 (1.04–1.38)			
								rs255105	0.043	0.0086		1.20 (1.05–1.38)			
							*CRHR1*	rs12942300	0.038	0.0086		1.31 (1.07–1.60)			
61	Binder EB et al. (2010) * [64]	746	1207	1953	740	649	*CRHBP*	rs10473984	0.0004	0.0006	N.A.	N.A.	Citalopram	6	9
								rs10474485	0.018	0.0062					
								rs10055255	0.005	0.0023					
							*CRHR1*	rs12942300	0.0087	0.0015					
							*CRHR2*	rs2267716	0.024	0.0098					
							*AVPR1A*	rs7307997	0.047	0.038					
62	Garriock HA et al. (2010) [65]	746	1207	1953	531	790	*OPRM1*	rs562859	N.A.	0.002	N.A.	1.33 (1.05–1.69)	Citalopram	6	9
								rs1323044	N.A.	0.003		1.46 (1.15–1.85)			
								rs540825	N.A.	0.003		1.37 (1.07–1.75)			
								rs658156	N.A.	0.003		1.54 (1.18–2.00)			
								rs13195018	N.A.	0.002		1.60 (1.23–2.08)			
								rs538174	N.A.	0.002		1.57 (1.20–2.05)			
								rs583664	N.A.	0.001		1.61 (1.23–2.10)			
								rs618207	N.A.	0.001		1.47 (1.13–1.91)			
63	Garriock HA et al. (2010) * [65]	746	1207	1953	531	669	*OPRM1*	rs562859	N.A.	0.002	N.A.	1.36 (1.06–1.74)	Citalopram	6	9
								rs1323044	N.A.	0.005		1.44 (1.12–1.85)			
								rs540825	N.A.	0.002		1.44 (1.12–1.86)			
64	Lin KM et al. (2011) [66] *	19	81	100	48	26	*ABCB1*	rs1882478	N.A.	0.037	N.A.	0.35 (0.17–0.71)	Escitalopram	8	8
								rs1045642		0.045	N.A.	0.34 (0.16–0.72)			
								rs10256836		0.021	N.A.	3.82 (1.58–9.22)			

N.A., data not available; M, male; F, female; FP, Follow-up period in weeks; OR, odds ratio; CI, confidence interval; Score, cumulative score for methodological quality assessment (see Tables S1 and S2 for detailed scoring) * Study name shows remission data. OR calculated using reported frequencies from the respective article. All the studies represent significant polymorphisms (*p* ≤ 0.05) with their corresponding genes.

**Table 2:** Characteristics of included genome-wide association studies concerning SSRI response in major depression patients.

No.	Study Name	Study Population	Responders (*n*)	Remitters (*n*)	Non-Remitters (*n*)	Non-Responders (*n*)	Total (*n*)	Genotyping Platform	SNPs Studied (*n*)
1	Myung W et al. (2015) [67]	Korean	497	312	558	373	870	Affymetrix Genome-Wide Human Single-Nucleotide Polymorphism (SNP) Array Chip 6.0	905,431
2	Biernacka JM et al. (2015) [68]	Asian, European	416	226	190	449	865	Illumina Human—Omni Express Exome Bead Chips	631,765
3	Hunter AM et al. (2013) [69]	European	869	N.A.	N.A.	247	1116	Affymetrix 500 K and 5.0 Human SNP Arrays	430,198
			Replication data set				706	Illumina Human 610 Quad Bead Chip	550,337
4	Tansey KE et al. (2012) [70]	European	N.A.	N.A.	N.A.	N.A.	2283	Illumina Human 610 Quad Bead Chips; Illumina Human 660 W-Quad Bead Chips	520,978
5	Ji Y et al. (2013) [71]	European	287	206	81	212	499	Illumina Human 610-Quad Bead Chips	550,337
6	Sasayama D et al. (2013) [72]	Japanese	61	N.A.	N.A.	31	92	Illumina Human CNV370 Quad Bead Chips	356,075
			Replication data set				136		
7	Uher R et al. (2010) [73]	European	N.A.	N.A.	N.A.	N.A.	706	Illumina Human 610 Quad Bead Chips	550,337

M, male; F, female. All the GWAS were performed on MDD patients having SSRIs as an antidepressant.

**Table 3:** Characteristics of genome-wide association studies of major depressive disorder.

No.	Study Name	Study Population	Cases (*n*)	Controls (*n*)	Total (*n*)	Platform	SNPs Studied (*n*)
1	Power RA et al. (2013) [74]	European	805	805	1610	Illumina 610 K bead array	457,670
2	Ripke S et al. (2013) [75]	European	9240	9519	18,759	Illumina 610 K, 317 K, 370 K, 550 K, Perlegen 600 K, Affymetrix 6.0	>200,000
			6783	50,695	57,478	N.A.	593
3	Wray NR et al. (2012) [76]	European	2431	3673	6104	Illumina 317 K, Illumina 370 K, Illumina 610 K, Affymetrix 600 K	657,366
4	Shi J et al. (2012) [77]	European	1020	1636	2656	Affymetrix 6.0	671,424
5	Shyn SI et al. (2011) [78]	European	1221	1636	2857	Affymetrix 6.0, 5.0 and 500 K, and Perlegen	500,568
6	Muglia P et al. (2010) [79]	European	1022	1000	2022	Illumina HumanHap550	551,101
			492	1052	1544	Affymetrix 5.0	370,697
7	Sullivan PF et al. (2009) [80]	European	1738	1802	3540	Perlegen	435,291
8	Rietschel M et al. (2010) [81]	European	604	1364	1968	Illumina HumanHap 550v3, and Illumina Human 610 W Quad Bead Chips	491,238
			409	541	950		

All the GWAS were performed on MDD patients vs. healthy controls.

## Supplementary Materials

Supplementary materials can be found at https://www.mdpi.com/1422-0067/20/8/1993/s1.
